# Cardiovascular and cerebrovascular diseases risk associated with the incidence of presenteeism and the costs of presenteeism

**DOI:** 10.1002/1348-9585.12167

**Published:** 2020-09-20

**Authors:** Koki Kimura, Tomohisa Nagata, Makoto Ohtani, Masako Nagata, Shigeyuki Kajiki, Yoshihisa Fujino, Koji Mori

**Affiliations:** ^1^ Department of Occupational Health Practice and Management Institute of Industrial Ecological Sciences University of Occupational and Environmental Health, Japan Kitakyushu Japan; ^2^ Data Science Center of Occupational Health University of Occupational and Environmental Health, Japan Kitakyushu Japan; ^3^ Department of Preventive Medicine and Community Health University of Occupational and Environmental Health, Japan Kitakyushu Japan

**Keywords:** absenteeism, coronary artery disease, cost, ischemic stroke, presenteeism, risk equation

## Abstract

**Objectives:**

The objective of this study was to estimate a risk of cardiovascular and cerebrovascular diseases for each worker and to determine whether this risk is associated with the incidence and costs of presenteeism, absenteeism, and medical/drug treatments.

**Methods:**

Established risk equations were used to estimate the 10‐year probability of developing coronary artery disease and ischemic stroke in male workers aged 40‐65 years who were recruited from four pharmaceutical companies in Japan. The incidence of presenteeism was defined as existence of presenteeism for the past a month, and the incidence of absenteeism was defined as existence of sick‐leave for the past three months by a self‐administered questionnaire. Each cost was calculated based on the human capital method. Data on medical/drug treatments were collected from health insurance claims.

**Results:**

The risks were calculated for 6047 workers. Individuals at moderate and high risk of coronary artery disease had a significantly higher rate of presenteeism and absenteeism than workers at low risk. Workers at moderate and high risk of ischemic stroke also had a significantly higher rate of presenteeism and absenteeism than workers at low risk. Mean costs for absenteeism and medical/drug treatments increased with the risk of developing coronary artery disease or ischemic stroke, while costs for presenteeism did not.

**Conclusions:**

To prevent the costs of presenteeism, workers not only at high risk but also at low and moderate risk of developing cardiovascular and cerebrovascular diseases should receive health care services.

## 1 INTRODUCTION

Employee illness results in both medical expenses and productivity loss from disability, absenteeism, and presenteeism. The role of health in productivity management has gradually garnered attention from government agencies and employers in Japan, where the working population is shrinking and aging.^1^ Studies have shown that a detailed breakdown of costs by medical condition is required for decision‐making in the field of employee health,^2^, ^3^ and we previously reported that the burden of presenteeism was greater than that of medical expenses and absenteeism in Japanese workers.^4^ The importance of health management measures targeting a decline in productivity that is attributable to presenteeism is becoming increasingly recognized in Japan.

Presenteeism has been associated with a stressful work environment^5^ and modulated by individual worker risk factors^6^ and the incidence of chronic illnesses.^7^ To reduce presenteeism, measures should incorporate both a population approach and a high‐risk approach. In the high‐risk approach, the employees at high risk of presenteeism should be identified and treated individually. In Japan, employers are obligated to conduct general periodic health examinations for all workers under the Industrial Safety and Health Act,^8^ while additional health assessments require cumbersome procedures to protect employees’ personal information.^9^ The general health examination therefore offers an effective opportunity to identify employees at high risk of presenteeism.

The costs of cardiovascular and cerebrovascular diseases constitute a great burden, and account for half of all causes of death and one‐quarter of work disability causes in the working population in Japan.^10^ Since employees are now expected to work more years than in the past because of the shrinking workforce, more individuals are projected to develop these diseases prior to retirement.^11^ Furthermore, the health effects of long working hours have become a major social issue, and cardiovascular and cerebrovascular diseases are regarded as the main adverse health effect of long working hours and industrial accidents.^12^ For these reasons, the Ministry of Health, Labour and Welfare has placed an emphasis on testing for and preventing these diseases.^13^

Studies have reported that medical and absenteeism costs increase with disease‐related risks,^14^, ^15^, ^16^, ^17^ and that the costs of presenteeism are also associated with a number of health risk factors and with the sum of risk factors identified in each employee.^7^, ^18^, ^19^, ^20^, ^21^, ^22^ These may also include non‐physical factors, such as the refusal to use safety belts or dissatisfaction with life.^22^ The costs of presenteeism may therefore not reflect the risk of cardiovascular and cerebrovascular diseases accurately. To the best of our knowledge, there are no published studies that correlate these risks with presenteeism. Such data would be an important resource for managing health and productivity in the workplace.

Risk equations have been developed to estimate the 10‐year probability of coronary artery disease and ischemic stroke in Japanese individuals.^23^ This probability can be calculated from age, sex, smoking status, systolic blood pressure, antihypertensive medication use, diabetes mellitus, and cholesterol levels; these data can be obtained from standardized laboratory tests and questionnaires during the general periodic health examinations. Coronary artery disease and ischemic stroke are major diseases in Japan. We hypothesized that employees at high risk of cardiovascular and cerebrovascular diseases, as estimated by the risk equations for coronary artery disease and ischemic stroke, would exhibit greater presenteeism than employees deemed at lower risk. We hypothesized as well that employees at high risk would exhibit greater absenteeism and medical/drug costs than employees deemed at lower risk. The purpose of this study was to examine the association between the 10‐year probability of developing these diseases and the incidence or costs of presenteeism, as well as the association with medical/drug treatment and absenteeism. By clarifying these associations, it is possible to estimate the effect and economic impact of preventing cardiovascular and cerebrovascular disease, and to clarify the characteristics of the target population for preventing presenteeism.

## 2 MATERIALS AND METHODS

We conducted a cross‐sectional study of male employees aged 40‐65 years in four pharmaceutical companies and their health insurance society. This age group was selected because the minimum age for the applicability of the risk equation method^23^ estimating the 10‐year probability of coronary artery disease and ischemic stroke is 40 years, and 65 is a retirement age. The predicted probability of incident coronary artery disease within 10 years and the predicted probability of ischemic stroke within 10 years were calculated for each employee by combinations of age, sex, smoking status, systolic blood pressure, cholesterol levels, antihypertensive medication use, and use of medications to control diabetes.^23^ These data were obtained from the standardized laboratory tests and health questionnaires administered to employees in 2014 in each participating company.

We divided the subjects into three groups according to the probability of incident coronary artery disease. We defined the workers whose probabilities were less than 0.5% as “low‐risk,” the workers whose probabilities were 0.5%–2.0% as “moderate‐risk,” and the workers whose probabilities were more than 2.0% as “high‐risk.” The cutoff points were set by reference to low‐density lipoprotein cholesterol management target‐setting.^24^ We also divided the probability of incident ischemic stroke into three categories using the same definition.

This study was approved by the ethics committee of the University of Occupational and Environmental Health, Japan, Kitakyushu, Japan (H26‐026 Date: 7/August/2019).

We designed a web‐based, self‐administered questionnaire about presenteeism and absenteeism in 2014. We asked participants whether they had experienced health issues at work over the preceding month. If the answer was yes, we asked whether the symptoms affected the quality and quantity of their work, in comparison with productivity during periods without symptoms. The quality and quantity were scored on a 0‐10 scale.^22^, ^25^ When participants had no health issues or indicated that their health issues had not affected the quality and quantity of their work at all, we defined the situation as “no presenteeism.” When participants indicated that their health issues did affect their work to any degree, we defined the situation as “presenteeism,” and the incidence of presenteeism was defined as existence of “presenteeism.” We calculated the presenteeism costs using the following formula^4^:

$$\text{Presenteeism costs}=\text{JPY}\;3,300\times 8\left(\text{working hours per day}\right)\times \left(1‐\text{quantity}\left(0‐10\right)\times \text{quality}\left(0‐10\right)/100\right)\times \left(\text{days }withrsymptomsrinraryear\right)$$

The mean payroll per person per hour was set at 3,300 Japanese Yen (JPY) and based on the average in large manufacturing companies in 2014 in Japan.^26^

We asked participants how many sick‐leave days they had taken over the preceding 3 months. All subjects of this study were full‐time employees, and they were guaranteed a sufficient sick leave according to their years of service. The salary was guaranteed by the company or health insurance unions depending on the number of days off. If the answer was none, we defined the situation as “no absenteeism.” All other answers were scored as “absenteeism,” and the incidence of absenteeism was defined as existence of “absenteeism.” We calculated the absenteeism costs using the following formula^4^:

$$\text{Absenteeism costs}=\text{JPY}\;3300\times 8\left(\text{working hours per day}\right)\times \left(\text{sick}‐\text{leave days in a year}\right)$$

We received inpatient medical and pharmaceutical claims, outpatient medical claims, and outpatient pharmaceutical claims data for all participants from the health insurance unions, which covered the period between 1 April 2014 and 31 March 2015. The claims did not include dental treatments or over‐the‐counter drug expenses. We defined the sum of inpatient medical and pharmaceutical claims, outpatient medical claims, and outpatient pharmaceutical claims as medical/drug costs. We excluded employees who spent more than 10 million JPY in medical expenses during that period to avoid the influence of catastrophic events.

### 2.1 Statistical analysis

We first calculated descriptive statistics (percentages, means, and standard deviation) in each risk category. Logistic regression was used to calculate the odds ratio of each incidence of presenteeism and absenteeism comparing each category of coronary artery disease and ischemic stroke risk. We calculated the odds ratio adjusted for occupation (categorical variables) and body mass index (continuous variable), which were not used for estimating the 10‐year probability of coronary artery disease and ischemic stroke. Next, we compared absenteeism, presenteeism, and medical/drug costs between the three categories. Statistical analysis was conducted using the Kruskal‐Wallis test, and post‐hoc multiple comparisons were made using the Mann‐Whitney U test with Bonferroni corrections. All tests were two‐tailed, with differences reported as significant if *P* < .05. All analyses were performed in SPSS version 25 (IBM SPSS, Armonk, NY, USA) and Stata version 16 (StataCorp, College Station, TX, USA).

## 3 RESULTS

We sent an e‐mail solicitation for questionnaires to 11 774 male employees aged 40‐65 years in four companies, and 6,581 individuals (56%) responded. We excluded 530 employees for missing data and four employees who experienced catastrophic events that required extensive medical treatment (more than 10 million JPY in medical expenses). The number of employees eligible for inclusion in the analysis was 6047. The numbers of employees in each coronary artery disease risk category (low, moderate, and high) were 2374, 1808, and 1865, respectively. The numbers of employees in each ischemic stroke risk category were 2319, 1628, and 2100, respectively. Table 1 lists the characteristics of study participants, stratified by each category.

**Table 1:** Demographic characteristics of the study population

	Total		Coronary artery disease risk						Ischemic stroke risk					
	N Mean	% SD	Low risk N = 1125		Moderate risk N = 4004		High risk N = 918		Low risk N = 2319		Moderate risk N = 3014		High risk N = 714	
			N Mean	% SD	N Mean	% SD	N Mean	% SD	N Mean	% SD	N Mean	% SD	N Mean	% SD
Age^a^														
40‐49	3,107	51	1,125	100	1,866	47	116	13	2,319	100	780	26	8	1
50‐59	2,679	44	0	0	2,022	50	657	72	0	0	2,183	72	496	69
60‐65	261	4	0	0	116	3	145	16	0	0	51	2	210	29
Occupation														
Clerical administrative support	830	14	148	13	559	14	123	13	299	13	436	14	95	13
Sales	2,507	41	403	36	1,643	41	461	50	970	42	1,213	40	324	45
Research and development	1,036	17	218	19	701	18	117	13	460	20	480	16	96	13
Production line	521	9	84	7	361	9	76	8	180	8	263	9	78	11
Other	1,153	19	272	24	740	18	141	15	410	18	622	21	121	17
Smoking^a^														
Yes	1,669	28	0	0	1,202	30	467	51	501	22	822	27	346	48
Treatment for hypertension^a^														
Yes	1,171	19	0	0	483	12	688	75	0	0	659	22	512	72
Treatment for diabetes mellitus^a^														
Yes	266	4	0	0	73	2	193	21	0	0	79	3	187	26
Treatment for hyperlipidemia^a^														
Yes	965	16	83	7	554	14	328	36	172	7	523	17	270	38
Body mass index (kg/m^2^)	23.9	3.2	22.9	2.5	23.8	3.1	25.7	3.6	23.2	2.8	24.1	3.2	25.2	3.5
Systolic blood pressure (mmHg)^a^	121	14	115	9	121	14	127	15	115	11	123	14	127	15
Diastolic blood pressure (mmHg)	77	10	73	8	78	10	82	11	74	9	79	10	81	11
Total cholesterol (mg/dL)	202	32	197	28	204	31	204	37	202	31	205	31	194	33
Low‐density lipoprotein cholesterol (mg/dL)	123	29	118	25	124	30	123	32	124	29	124	29	114	29
High‐density lipoprotein cholesterol (mg/dL)	58	14	60	13	59	15	53	14	58	14	59	15	55	14
Triglycerides (mg/dL)	127	99	97	50	124	84	175	165	114	74	130	106	155	126
Fasting blood glucose (mg/dL)	97	16	92	8	96	14	108	27	93	10	97	15	109	27
Glycated hemoglobin (HbA1c, %)	5.5	0.6	5.3	0.3	5.4	0.5	5.9	0.9	5.3	0.4	5.5	0.6	5.9	0.9

^a^ These variables were used to calculate the risk of coronary artery disease and ischemnic stroke.

Regarding coronary artery disease, the incidence of presenteeism (defined as existence of presenteeism for the past a month) was recorded for 19%, 22%, and 27% of participants at low, moderate, and high risk, respectively (Table 2). The incidence of absenteeism (defined as existence of sick‐leave for the past three months) was recorded for 17%, 21%, and 30% of participants at low, moderate and high risk, respectively. Workers at moderate and high risk also had a significantly higher risk of incidence of presenteeism than workers at low risk (OR: 1.18; CI: 1.00‐1.40 for moderate‐risk workers and 1.46 [CI: 1.18‐1.81] for high‐risk workers). Workers at moderate and high risk had a significantly higher risk of incidence of absenteeism than workers at low risk (odds ratio [OR]: 1.29; 95% confidence interval [CI]: 1.08‐1.53 for moderate‐risk workers and 2.11 [CI: 1.69‐2.63] for high‐risk workers).

**Table 2:** Adjusted odds ratio of incidence of presenteeism and absenteeism by risk category

	Incidence of presenteeism^a^				Incidence of absenteeism^b^			
	Proportion (%)	Adjusted odds ratio^c^	95% confidence interval	*p* value	Proportion (%)	Adjusted odds ratio^c^	95% confidence interval	*P* value
Coronary artery disease								
Low risk	19	ref			17	ref		
Moderate risk	22	1.18	1.00‐1.40	.048	21	1.29	1.08‐1.53	.005
High risk	27	1.46	1.18‐1.81	<.001	30	2.11	1.69‐2.63	<.001
Ischemic stroke								
Low risk	20	ref			19	ref		
Moderate risk	23	1.17	1.02‐1.33	.022	22	1.23	1.07‐1.41	.003
High risk	25	1.24	1.01‐1.51	.039	31	1.94	1.59‐2.36	<.001

^a^ The incidence of presenteeism was defined as existence of presenteeism for the past a month by a self‐administered questionnaire.

^b^ The incidence of absenteeism was defined as existence of sick‐leave for the past three months by a self‐administered questionnaire.

^c^ Adjusted for occupation (categorical variables) and body mass index (continuous variable).

Regarding of ischemic stroke, the incidence of presenteeism was recorded for 20%, 23%, and 25% of participants at low, moderate, and high risk, respectively. The incidence of absenteeism was recorded for 19%, 22%, and 31% of participants at low, moderate and high risk, respectively. Workers at moderate and high risk also had a significantly higher risk of incidence of presenteeism than workers at low risk (OR: 1.17; CI: 1.02‐1.33 for moderate‐risk workers and 1.24 [CI: 1.01‐1.51] for high‐risk workers). Workers at moderate and high risk of ischemic stroke had a significantly higher risk of incidence of absenteeism than workers at low risk (OR: 1.23; CI: 1.07‐1.41 for moderate‐risk workers and 1.94 [CI: 1.59‐2.36] for high‐risk workers).

**Figure 1:**
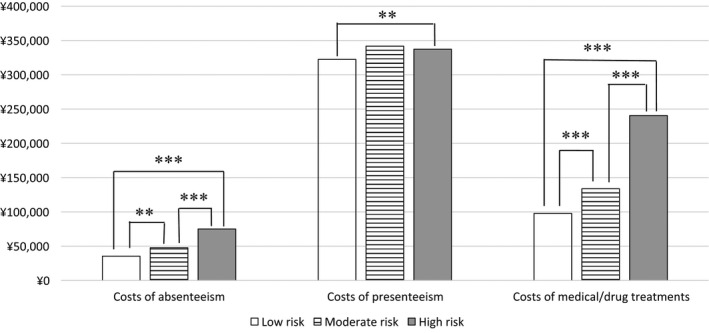
Mean costs of absenteeism, presenteeism, and medical/drug treatments per person per year (Japanese Yen in 2014) stratified by risk of coronary artery disease. **P < .01; ***P < .001

The mean presenteeism costs per person per year in groups at low, moderate, and high risk for coronary artery disease were JPY 322 418, JPY 341 768, and JPY 337 277, respectively (Figure 1). Only the high‐risk and low‐risk groups differed significantly (*P* < .01). The mean absenteeism costs per person per year in groups at low, moderate, and high risk for coronary artery disease were JPY 35 388, JPY 47 697, and JPY 75 059, respectively. All risk groups differed significantly (*P* < .001). Mean medical/drug costs per person per year in the high‐risk group were highest (JPY 240 486), followed by costs in the moderate‐risk group (JPY 133 820) and in the low‐risk group (JPY 97 816). There was a significant cost difference between all risk groups (*P* < .001).

**Figure 2:**
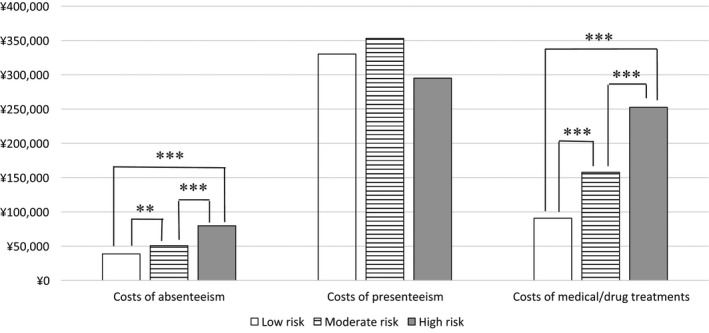
Mean costs of absenteeism, presenteeism, and medical/drug treatments per person per year (Japanese Yen in 2014) stratified by risk of ischemic stroke. **P < .01; ***P < .001

The mean presenteeism costs per person per year in groups at low, moderate, and high risk for ischemic stroke were JPY 330,213, JPY 353 118, and JPY 295 122, respectively (Figure 2). The three risk groups differed significantly in the Kruskal‐Wallis test (*P* = .031) but not in the post‐hoc multiple comparisons test (Mann‐Whitney U‐test with Bonferroni correction). The mean absenteeism costs per person per year in groups at low, moderate, and high risk for ischemic stroke were JPY 38 729, JPY 50 733, and JPY 79 792, respectively. All risk groups differed significantly. Mean medical/drug costs per person per year in the low‐, moderate‐, and high‐risk groups were JPY 90 838, JPY 157 818, and JPY 252 531, respectively. The three groups differed significantly (*P* < .001).

## 4 DISCUSSION

We sought to associate the probability of developing coronary artery disease and ischemic stroke with the incidence of presenteeism and absenteeism, as well as with the annual mean cost per employee of presenteeism, absenteeism, and medical/drug treatments. Presenteeism and absenteeism were higher in workers at moderate and high risk of coronary artery disease and ischemic stroke. The mean costs of absenteeism and medical/drug treatments were higher in the high‐risk groups, and presenteeism costs were higher in the group at high risk for coronary artery disease but not ischemic stroke. Overall, the costs of presenteeism were much higher than those of absenteeism or medical/drug treatments, which is consistent with the findings of our previous study.^4^ As far as we know, this is the first study to assess the association between risks of cardiovascular and cerebrovascular diseases calculated with risk equations and presenteeism, absenteeism, and medical/drug treatment simultaneously.

Our findings of increased absenteeism and medical/drug treatments in the high‐risk group are in accordance with those of studies that reported that absenteeism or medical/drug costs increased as the number of cardiometabolic risk factors increased,^14^, ^15^, ^16^, ^17^ and suggest that employers would benefit from reducing absenteeism and medical/drug treatments to intervene in high‐risk individuals identified from health examinations.

The percentage of employees displaying symptoms that cause presenteeism is reported to be approximately 16%–22%^4^, ^27^ among workers in large Japanese companies. Therefore, identifying individuals at high risk and offering them treatment measures would constitute an effective approach. In Japan, employers are required to implement intervention measures in occupational health after general health examination. Since studies have shown that presenteeism increases as the number of risk factors for cardiovascular and cerebrovascular diseases increases,^7^, ^18^, ^19^, ^20^, ^21^, ^22^ we examined the feasibility of screening individuals at high risk of presenteeism using the disease risk factors that can be identified by the general health examination. We found that presenteeism was higher in individuals at high risk of coronary artery disease and ischemic stroke, suggesting that this approach is effective. This result contradicts the fact that costs of presenteeism were not higher according to rising the risk of coronary artery disease and ischemic stroke. Presenteeism costs are calculated not only by the existence of preesnteeism but also by factors that affect the work. A past study revealed that the highest cost burdens of presenteeism from chronic illness were related to mental (behavioral) health conditions.^4^ Although the rate of co‐morbidity between cardiovascular and cerebrovascular disease and depression is relatively high at around 20%,^28^ the risk factors for those diseases are not exactly the same. There might be many people with mental health illness in the low or moderate risk group of cardiovascular and cerebrovascular diseases as those in the high‐risk group. In addition to this, the amount of presenteeism costs for people with symptoms of mental illness is higher than for other symptoms.^4^ For that reason, costs of presenteeism in workers at low and moderate risk for both coronary artery disease and ischemic stroke may contain costs of presenteeism related to mental health conditions. Costs of presenteeism in workers at low and moderate risk should not be neglected since it involves much higher than costs of absenteeism or medical/drug. These findings also indicate that employers should consider investing in countermeasures against presenteeism, such as interventions to relieve stressful conditions in the workplace.

This study has several limitations. First, we used data from employees of large pharmaceutical companies. The smoking rate was 28% among the subjects of this study (mostly 40‐59 year old men). According to the National Health and Nutrition Survey,^29^ which is a representative sample of the general population in Japan, smoking rate was 44.2% among men in their 40s and 36.4% in men in their 50s in the 2014 survey. The subjects in this study may be healthier than the general population. However, since this study calculates the absolute risk of cardiovascular and cerebrovascular diseases using data from lifestyle and blood tests, the results of this study can be used in other groups. The future study is needed to confirm reproducibility of this results in small and medium enterprises and other industries. This study was conducted only among men, and it will be necessary to consider it among women in the future. Second, we could not calculate the costs of over‐the‐counter drugs, but as sales of such drugs in Japan total only JPY 94 billion, compared with JPY 985 billion for prescription drugs,^30^ we estimate that the influence of the former is small. Third, dental claims were not included in the medical/drug costs. Again, as dental costs are estimated at only 6.8% of total expenditure for medical care,^31^ we surmise that their influence on our findings is negligible. Fourth, this study was cross‐sectional, so we were unable to estimate whether programs aimed at preventing cardiovascular and cerebrovascular diseases reduce the economic burdens of presenteeism, absenteeism, and medical/drug treatments. Determining the efficacy of prevention programs would require intervention studies.

Despite the limitations, our study adds to the literature assessing health‐related costs in the context of cardiovascular and cerebrovascular diseases. The findings could assist employers in developing effective strategies for the promotion of workplace health and human capital, especially in Japan.

## DISCLOSURE

*Approval of the research protocol*: This study was approved by the ethics committee of the University of Occupational and Environmental Health, Kitakyushu, Japan, and was conducted in full accordance with the World Medical Association Declaration of Helsinki. *Informed consent*: We explained the study protocol and obtained opt‐out consent. *Registry and the registration no. of the study/trial*: N/A. *Animal Studies*: N/A. *Conflict of interest*: None declared.

## AUTHOR CONTRIBUTIONS

TN and KM conceived and coordinated the project. KK, TN, and MO completed the data analysis. KK and TN drafted the initial manuscript. MN, S.K, YF, and KM revised the manuscript. All authors commented on drafts of the report.
